# The case for investing in provider-administered subcutaneous DMPA: a costing study

**DOI:** 10.1136/bmjgh-2024-018761

**Published:** 2025-10-22

**Authors:** Holly M Burke, Wingston Felix Ng'ambi, Rick Homan, Megan M Lydon, Jessie Salamba Chirwa, Hannah Kachule, Grace Jabu, Mario Chen

**Affiliations:** 1 Contraceptive Research, Development, and Introduction, FHI 360, Durham, North Carolina, USA; 2 Data Science and Statistical Consulting Center, Lilongwe, Malawi; 3 FHI 360, Durham, North Carolina, USA; 4 Reproductive, Maternal, Newborn and Child Health Division, FHI 360, Durham, North Carolina, USA; 5 Reproductive Health Directorate, Government of Malawi Ministry of Health, Lilongwe, Malawi; 6 Biostatistics, FHI 360, Durham, North Carolina, USA

**Keywords:** Health economics, Health systems, Health services research, Cohort study, Health policy

## Abstract

**Introduction:**

Depot medroxyprogesterone acetate (DMPA) is available as provider-administered intramuscular (DMPA-IM) and subcutaneous injections (PA DMPA-SC), as well as DMPA-SC self-injection (SI), which enhances accessibility and continuation by reducing facility visits. However, DMPA-SC has a higher unit price than DMPA-IM, and low SI uptake can undermine its cost-effectiveness. This study assessed whether PA DMPA-SC’s potential benefits justify its higher cost compared with DMPA-IM in Malawi, an early SI adopter with a higher proportion of SI.

**Methods:**

In this costing study, we used recursive Markov models to examine 12-month service use patterns and costs for each injectable option. We collected data on supplies and infrastructure supporting injectable services (by product and location: facility or community) through site assessments and group discussions with facility-based providers and community health workers across nine facilities. We derived transition and discontinuation probabilities from longitudinal surveys about service use and experiences in a concurrent cohort study of injectable users aged 15–49.

**Results:**

We conducted 23 provider group discussions and enrolled 992 cohort participants. PA DMPA-SC users were twice as likely to discontinue their method than DMPA-IM users (HR 2.01 (1.66–2.43)). However, a significantly higher proportion of PA DMPA-SC users transitioned to SI (47.1%), and did so faster, compared with DMPA-IM users (20.4%).

The annual DMPA service cost per person-year of use was not substantially different between DMPA-IM (US$11.01) and PA DMPA-SC (US$11.47). Community-based services were 31% lower in cost at US$8.07 than facility-based services at US$11.61.

In facilities, DMPA-SC is less expensive than DMPA-IM when 40% or more of all DMPA-SC visits are for SI; for community-based services, the threshold is 23%. Service volume data indicate these thresholds are achievable in Malawi.

**Conclusions:**

Not all DMPA-SC users need to self-inject for DMPA-SC to be cost-competitive with DMPA-IM. Use of PA DMPA-SC may increase the uptake of SI over time.

**WHAT IS ALREADY KNOWN ON THIS TOPIC**

- Depot medroxyprogesterone acetate subcutaneous (DMPA-SC) self-injection is known to be cost-effective, but provider-administered DMPA-SC has a higher unit cost than DMPA-IM, raising concerns about its value. Evidence is lacking on whether the potential benefits of provider-administered DMPA-SC justify its continued use and scale-up over DMPA-intramuscular (IM).

**WHAT THIS STUDY ADDS**

- This study found that in Malawi, women receiving provider-administered DMPA-SC transitioned to self-injection more often and more quickly than those receiving DMPA-IM. As a result, provider-administered DMPA-SC achieved cost comparability with DMPA-IM, especially when provided in community settings. The findings suggest that even partial uptake of self-injection can make DMPA-SC cost-competitive.

**HOW THIS STUDY MIGHT AFFECT RESEARCH, PRACTICE OR POLICY**

- Supporting provider-administered DMPA-SC alongside self-injection could expand contraceptive choice and accelerate self-injection uptake. National programmes should offer all three injectable options—DMPA-IM, provider-administered DMPA-SC and self-injection—across service delivery channels. These findings can guide policy decisions in Malawi and inform global strategies for scaling up DMPA-SC.

## Introduction

### Background

Depot medroxyprogesterone acetate (DMPA) remains a predominant form of family planning in sub-Saharan Africa and the most used method in Malawi, representing 52% of the contraceptive method mix.^1^ It is available in three forms: provider-administered intramuscular injection (DMPA-IM), provider-administered subcutaneous injection (PA DMPA-SC) and self-injected (SI) DMPA-SC. Both DMPA-IM and DMPA-SC offer 3 months of pregnancy prevention, but DMPA-SC is packaged in a prefilled unit that allows for SI or provider-administration. Its ease of use and potential for advanced provision can reduce clinic visits, lowering costs to the healthcare system and clients and increasing accessibility and continuation of the method.^2–6^

Currently, DMPA-SC is only available as Pfizer’s branded product called Sayana Press, making it more expensive than generic DMPA-IM.^7–9^ Nonetheless, studies conducted in Burkina Faso, Senegal and Uganda found SI of DMPA-SC cost-effective for women and health systems when compared with facility-based health worker administration of DMPA.^10–13^

Despite these advantages, uptake of SI remains low—at least 80% of DMPA-SC use is provider-administered.^14^ Barriers include supply chain issues (stockouts, understocking and imminent expiration which is conducive for provider-administered injectables but not advanced provision for SI), lack of provider training, low community awareness and client hesitancy.^14–18^ This undermines the cost-effectiveness gains associated with SI.

Over 55 countries have registered or are scaling up DMPA-SC for SI.^8^ However, concerns persist among governments and donors about investing in a higher-cost product if it is primarily provider-administered because of the assumption that this undermines its cost-effectiveness. While cost-effectiveness is important, offering more methods, including modes of administration, promotes overall uptake and continuation of family planning.^19^

At the same time, there is an urgent need to assess whether provider-administered DMPA-SC offers benefits that justify its higher cost compared with DMPA-IM and to determine the proportion of DMPA-SC users who must self-inject for DMPA-SC to be cost-competitive. Further, previous costing studies were conducted in pilot settings with limited generalisability. This study addresses these gaps by evaluating routine service delivery in Malawi’s public sector, which currently has the most developed SI markets among low-resource countries.^20^

Malawi was among the first to introduce SI and had one of the highest proportions of DMPA-SC self-injected—27% at the time of data collection.^21^ From 2015 to 2017, FHI 360, a global nonprofit human development organization (formerly Family Health International), conducted a randomised controlled trial in Malawi that demonstrated higher continuation rates among self-injectors of DMPA-SC compared with provider-administered DMPA-SC users, promoting rapid national scale-up in 2018.^4 22^ Evidence from other countries supports this finding.^5^ Unlike other countries that introduced provider-administered DMPA-SC first, Malawi offered both provider-administered and SI options simultaneously—a strategy that may explain its relatively advanced market. Still, uptake of SI remains lower than expected.

Findings from this study will inform Malawi’s family planning programme and global best practices for introducing and scaling up DMPA-SC and SI.

### Objectives

The study aimed to compare user experiences with provider-administered DMPA-IM, provider-administered DMPA-SC and SI DMPA-SC over 12 months. The secondary objective was to compare the resources required to offer each option. Specifically, we asked:

1. What is the ‘value proposition’ of each injectable option in terms of user continuation and health system efficiency, and does this compensate for the higher unit price of DMPA-SC?
2. What is the ‘value proposition’ of community-based versus facility-based distribution?

## Methods

### Study design

This was a costing study that used longitudinal data from a cohort study on injectable user experience and cross-sectional data on resources and associated costs to deliver DMPA services.

### Study setting

The study was conducted in nine public facilities and their surrounding catchment areas across five districts in Malawi’s Central and Southern regions: Lilongwe, Dowa, Nkhotakota, Nsanje and Thyolo. Facilities were purposively selected in collaboration with the Ministry of Health and the Delivering Innovation in Self-Care (DISC)-Malawi project,^23^ prioritising sites that consistently offered all three injectable options and had community health workers (CHWs) trained or scheduled for training to deliver these methods. During the study, DISC supported demand creation, provider training using its ‘Moment of Truth’ SI curriculum and supply chain support for DMPA-SC.^15 24^

### Cohort participants and data collection

A prospective cohort of new and continuing injectable users, aged 15–49, seeking injectables at the study sites were recruited into the study. Participants were surveyed approximately every 3 months over a 12-month period. The study aimed for a sample that included approximately one-third of each DMPA-IM, provider-administered DMPA-SC and SI users. Additional eligibility criteria included the intent to remain in the study area for the duration of the study, willingness to participate in follow-up and consent to provide contact information.

Participants were recruited by facility-based family planning providers (eg, nurses and midwives) and CHWs called health surveillance assistants (HSAs) in Malawi, some of whom temporarily worked in facilities due to staffing shortages.

Using a recruitment script approved by the institutional review boards (IRBs), the providers told potentially eligible participants about the study after the clients selected their family planning method and completed their services according to the Ministry of Health service delivery guidelines. According to the guidelines, clients are counselled on all methods, and if they select injectables, then the clients choose one of the three options (DMPA-IM, provider-administered DMPA-SC or SI). Interested clients were referred to female study staff. The staff, who were trained in ethics and the protocol, briefly introduced the study to potential participants before asking if they could relocate to another location near the service delivery site that allowed for audio privacy. In this new location, staff explained the study in detail and went through the informed consent process. All participants provided written informed consent before data collection. We obtained a waiver of parental consent from the IRBs for participants under 18 years of age to protect minors’ privacy regarding their family planning use. Although literacy was not formally assessed, illiterate participants were asked to identify an impartial witness—who was not affiliated with the study—to be present during the informed consent process. This witness confirmed that all information on the consent form was provided verbally to the participant.

For the longitudinal cohort study, participants individually responded to a predefined set of quantitative survey questions. The structured surveys measured demographics, family planning method use and satisfaction and experiences with method access and use, including side effects. The surveys were programmed in KoboToolBox, and study staff collected participants’ responses on password-protected tablets using KoboCollect. Their answers were recorded as numerical values and categorical data. To minimise data entry errors, the surveys were designed with programmed logic that included restricted value ranges, logic checks and automated warnings for incomplete data fields.

Study staff conducted four follow-up surveys approximately every 3 months at a private location of the participants’ choosing. Follow-up surveys started approximately 4 months postenrolment to avoid influencing reinjection behaviour. All participants were followed up for the full 12 months, even if they stopped using injectables or family planning altogether. Participants were reminded at each survey of their voluntary participation and that participation would not affect the services they received. Surveys were conducted in English or Chichewa, and participants received 6000 Malawian Kwacha (MWK) (approximately US$5.00) per survey.

Female study staff conducted the enrolment and follow-up surveys to enhance participant comfort. Whenever possible, the same study staff surveyed the same participants for the duration of the study to foster rapport. Study staff training included classroom instruction and pretesting the procedures with similar clients to ensure readiness for data collection.

Participant names and contact information were stored separately from all survey data to ensure deidentification. The electronic data were uploaded daily to a secure, password-protected server or as soon as an internet connection became available. All signed informed consent forms were kept in locked cabinets. Access to both the deidentified data and the consent forms was limited to a small number of authorised study staff whose duties required it.

### Provider participants and data collection

To compare the resources required for each injectable option (secondary objective), we conducted a resource requirements analysis in the same facilities and catchment areas as our concurrent cohort study. This analysis took a whole-of-society perspective, including costs at the client, provider, facility and health system levels.

Data were collected through group discussions with DMPA service providers (nurses, midwives and HSAs) and guided by a structured protocol and data collection tool. Discussions focused on provider experiences and time spent delivering DMPA-IM or DMPA-SC (via provider administration, supervised SI or advance provision for SI). Providers were eligible to participate if they were 18 years or older and active DMPA service providers in the study facilities or surrounding communities. We conducted separate discussions with facility-based providers and HSAs. Providers were further grouped by SI experience, based on whether they had been trained within the past 2 years.

To complement provider input, study staff conducted site assessments to document infrastructure (ie, size of the physical space used), equipment (eg, exam table, chair) and supplies (eg, gloves, gauze, bandages) used in DMPA service delivery by product type and location (facility or community). Resources were recorded using a standardised Excel template. No client observations were performed.

The facility-in-charge and/or other supervisors at the study facilities informed potentially eligible providers at their facility and catchment areas about the study using an IRB-approved recruitment script. The script briefly explained the study, the procedures, and had a clear statement that the providers can refuse to participate, and their decision will not affect their employment.

The discussions were conducted in quiet private locations at or near the study facility to ensure privacy and confidentiality. Study staff, trained in ethics and the protocol, obtained written informed consent and confirmed eligibility from each provider individually before conducting the group discussions. All provider participants were literate, as this was a requirement of their profession. Discussions were held in English or Chichewa. The goal was to engage providers in a discussion of the overall process of providing DMPA services to clients while documenting key factors related to time spent with the client and how that time changes depending on where the service is being provided (ie, facility or community), the product being used (DMPA-IM or DMPA-SC) and the process of administration (eg, provider-administered, SI, new client, existing client). Time estimates were recorded on flip charts during the discussion and then photographed after the discussion ended. Study staff entered these quantitative data into a standardised Excel template for analysis. Discussions were also audio-recorded and transcribed for documentation (not analysis). Participants received MWK6000 (approximately US$5.00) and refreshments.

To ensure confidentiality, all participant names and contact information were stored separately from their data. Electronic data were secured with passwords, while the signed informed consent forms were stored in locked cabinets. Access to both the deidentified data and the consent forms was limited to a small number of authorised study staff whose duties required it.

### Sample sizes

To enable robust statistical comparisons across multiple outcomes, we aimed for the largest feasible cohort sample size. Power calculations focused on continuation rates, using conservative assumptions informed by prior DMPA-SC studies. In our previous Malawi study, the 12-month continuation was 73% for SI and 45% for provider-administered DMPA-SC (84% vs 53% using a less stringent definition), with a >20% loss-to-follow-up.^4^

Assuming 70% continuation in the SI group and 59% continuation in the DMPA-IM or provider-administered DMPA-SC group (HR of 1.5 for the risk of discontinuation) and a 20% loss-to-follow-up, we estimated a sample of 900 injectable users—300 per group (DMPA-IM, provider-administered DMPA-SC and SI)—were needed to detect differences with 80% power. Calculations used a time-to-event (logrank) analysis to account for the discontinuation time and censored times (eg, lost to follow-up) as implemented by the Power Analysis and Sample Size 2022 software.^25 26^

For the resource analysis, we planned three group discussions per facility to capture perspectives from facility-based providers, experienced HSAs and newly trained HSAs. This sample size was based on evidence that 80% thematic is typically achieved with three to six focus groups.^27^ We aimed to have between six and 10 providers in each group discussion.

### Patient and public involvement

The Malawi Ministry of Health’s Reproductive Health Directorate contributed to study design, implementation, interpretation and dissemination. Family planning implementing partners, providers and clients in Malawi were central to the dissemination of the study findings.

### Data analyses

We assessed attrition bias in the cohort using t-tests and χ^2^ tests across the following variables: district, age, religion, marital status, parity, education, employment status and covert family planning user status. Primary analyses focused on continuation rates, user satisfaction, method switching and resource use (eg, out-of-pocket costs and time spent seeking services) over 12 months. Descriptive statistics, Kaplan-Meier probability curves and log-rank tests were used to compare continuation and transition to SI across the three injectable options. ‘Method status change’ included switching to a different DMPA product or a different family planning method as well as discontinuing family planning use altogether. Transitions to SI were defined as moving from enrolment method to SI and continuing with SI for the remainder of the study or until lost to follow-up. Censoring occurred when follow-up data were missing or participants no longer needed family planning. Analyses were conducted in STATA Statistical Software (18.5 Standard Edition).

Our main costing analyses focused on DMPA service delivery costs within the healthcare system. This included the commodities, supplies, labour and infrastructure. We compared groups within the main cohort study (DMPA-IM vs provider-administered DMPA-SC or facility-based services vs community-based services) over a 1-year period, noting changes in the type of DMPA service used for those who continue to use DMPA or switch to non-DMPA methods or discontinue. In both cases, applying an intent-to-treat approach, we kept clients in the cohorts to which they were originally assigned (no crossovers). Thus, if clients move from their initial provider-administered form of DMPA to the alternative DMPA product or become self-injectors, those clients and their associated costs remain within the model for which they began. Likewise, for clients who began receiving services in one location but switched to the alternative location, rather than crossing over into the alternative model, they remain, and their associated costs continue to accumulate in their initial model. These parallel models allow for the most direct comparison of the two cohorts over time. This comparison only considers the DMPA service costs without any costs associated with switching to less effective methods or the costs of unintended pregnancies.

We employed recursive (quarterly) Markov models to simulate service use and costs over 12 months. When comparing the two cohorts, we began with a simulated group of 100 000 persons in each cohort at Time 0. We also introduced some variability using a beta distribution for some of the inputs, as they were estimates rather than fixed parameters. We then applied a Monte Carlo simulation of 1000 cohorts, noting the mean of the metric of interest and the fifth and 95th percentiles to be interpreted as 95% CIs for illustrative purposes.

The model inputs included published contraceptive method failure rates,^28^ probabilities of transition and discontinuation probabilities from the cohort and service delivery costs from the provider discussions and site assessments. Provider time and non-labour inputs were valued based on estimates from the Guttmacher Institute.^29^ DMPA-SC was priced US$0.20 higher than DMPA-IM to reflect a situation where there is no price guarantee and no generic available. Our models reflect the observed behaviour of persons who started with DMPA-IM or provider-administered DMPA-SC and where they migrated during the 1-year follow-up. To clarify, at time 0, we did not use data from the SI cohort. However, as DMPA-IM and provider-administered DMPA-SC users adopted SI in subsequent periods, we used data from the SI cohort to inform estimates of retention or transition. We also calculated the break-even proportion of SI needed for DMPA-SC to be less costly per person-year of use than DMPA-IM. Models were run in Excel (V.2503).

To complement these analyses, we extracted and tallied deidentified monthly injectable service volume data for the study facilities during the 12 months of the cohort study. These data are routinely collected by the Ministry of Health family planning providers using paper registers and then are entered monthly into the Health Management Information System by the Ministry of Health data clerks at the facilities. In addition, we asked the data clerks at each study facility to maintain a separate count of deidentified DMPA service provision by the community-based HSAs and the facility-based providers with details on whether the DMPA-SC was provider-administered or SI. These data are already collected at the facility and HSA level, but are routinely aggregated prior to reporting into the Health Management Information System, causing these details to be lost.

## Results

### Description of cohort participants

From April to May 2023, we enrolled 992 women into the cohort: 393 DMPA-IM users, 299 provider-administered DMPA-SC users and 300 SI users. Most participants (87%) were enrolled in health facilities (table 1).

**Table 1:** Participant characteristics at enrolment

Participant characteristic	Overall (n=992) n (%)	DMPA-IM (n=393) n (%)	Provider-administered DMPA-SC (n=299) n (%)	Self-injection (n=300) n (%)
Enrolment location				
Health facility	864 (87.1)	369 (93.9)	291 (97.3)	204 (68)
HSA/community	128 (12.9)	24 (6.1)	8 (2.7)	96 (32)
Age, mean (SD) (range)	27.8 (6.6) (16–49)	27.9 (6.5) (16–49)	28.4 (6.7) (16–48)	27.1 (6.7) (16–47)
Age categories:				
15–17	9 (0.9)	3 (0.8)	3 (1)	3 (1)
18–24	372 (37.5)	142 (36.1)	99 (33.1)	131 (43.7)
25–49	611 (61.6)	248 (63.1)	197 (65.9)	166 (55.3)
Education				
None	60 (6.1)	26 (6.6)	17 (5.7)	17 (5.7)
Some primary school	401 (40.4)	174 (44.3)	105 (35.1)	122 (40.7)
Completed primary school	111 (11.2)	44 (11.2)	29 (9.7)	38 (12.7)
Some secondary school	208 (21)	73 (18.6)	71 (23.8)	64 (21.3)
Completed secondary school	165 (16.6)	59 (15)	62 (20.7)	44 (14.7)
Some postsecondary	20 (2)	9 (2.3)	6 (2)	5 (1.7)
Completed postsecondary	27 (2.7)	8 (2)	9 (3)	10 (3.3)
Religion				
Christian	865 (87.2)	360 (91.6)	263 (88)	242 (80.7)
Muslim	124 (12.5)	32 (8.1)	35 (11.7)	57 (19)
None	2 (0.2)	1 (0.3)	1 (0.3)	0 (0)
Other	1 (0.1)	0 (0)	0 (0)	1 (0.3)
Worked for pay outside the home within last year				
Yes	302 (30.4)	114 (29)	113 (37.8)	75 (25)
No	690 (69.6)	279 (71)	186 (62.2)	225 (75)
Multidimensional Poverty Index^30^				
Mean score (SD)	0.3 (0.2)	0.3 (0.2)	0.3 (0.2)	0.3 (0.2)
Proportion in severe poverty n (%)	392 (42.8)	160 (43.0)	96 (34.2)	136 (51.5)
Ever given birth				
Yes	976 (98.4)	389 (99)	295 (98.7)	292 (97.3)
No	16 (1.6)	4 (1)	4 (1.3)	8 (2.7)
Among those who have ever given birth, no. of living children (n=976), mean (SD) (range)	2.3 (1.3) (0–7)	2.3 (1.3) (0–7)	2.3 (1.2) (0–7)	2.2 (1.4) (1–7)
Fertility intention (n=992)				
Would not like a child/any more children	366 (36.9)	153 (38.9)	106 (35.5)	107 (35.7)
Would like a/another child in more than 2 years	445 (44.9)	159 (40.5)	138 (46.2)	148 (49.3)
Would like a/another child within 2 years	170 (17.1)	75 (19.1)	53 (17.7)	42 (14)
Don’t know	11 (1.1)	6 (1.5)	2 (0.7)	3 (1)
Married				
Yes	795 (80.1)	316 (80.4)	241 (80.6)	238 (79.3)
No	197 (19.9)	77 (19.6)	58 (19.4)	62 (20.7)
Husband/partner knows about family planning appointment (n=971)				
Yes	858 (88.4)	336 (87.5)	261 (89.1)	261 (88.8)
No	113 (11.6)	48 (12.5)	32 (10.9)	33 (11.2)
Experience with family planning				
New user	95 (9.6)	47 (12)	24 (8)	24 (8)
Prior user	897 (90.4)	346 (88)	275 (92)	276 (92)
Contraceptive methods ever used:				
Tubal ligation	0 (0)	0 (0)	0 (0)	0 (0)
Vasectomy	0 (0)	0 (0)	0 (0)	0 (0)
Copper IUD	13 (1.3)	6 (1.5)	4 (1.3)	3 (1)
Hormonal IUD	15 (1.5)	2 (0.5)	10 (3.3)	3 (1)
Injectables (any)	481 (48.5)	192 (48.9)	167 (55.9)	122 (40.7)
DMPA-IM	394 (39.7)	158 (40.2)	131 (43.8)	105 (35)
DMPA-SC provider-administered	161 (16.2)	36 (36)	99 (33.1)	26 (8.7)
Self-injection	112 (11.3)	35 (8.9)	36 (12)	41 (13.7)
Implant	259 (26.1)	89 (22.7)	100 (33.4)	70 (23.3)
Pills	225 (22.7)	84 (21.4)	87 (29.1)	54 (18)
Emergency contraception	31 (3.1)	7 (1.8)	14 (4.7)	10 (3.3)
Male condom	316 (31.9)	129 (32.8)	93 (31.1)	94 (31.3)
Female condom	23 (2.3)	9 (2.3)	7 (2.3)	7 (2.3)
Diaphragm/spermicide	4 (0.4)	2 (0.5)	1 (0.3)	1 (0.3)
Lactational amenorrhoea	46 (4.6)	19 (4.8)	18 (6)	9 (3)
Standard days	50 (5)	19 (4.8)	24 (8)	7 (2.3)
Withdrawal	50 (5)	15 (3.8)	27 (9)	8 (2.7)
Other	2 (0.2)	393 (100)	1 (0.3)	1 (0.3)

DMPA-IM, depot medroxyprogesterone acetate-intramuscular; DMPA-SC, depot medroxyprogesterone acetate-subcutaneous; HSA, health surveillance assistant; IUD, intrauterine device.

The average age was 28 years, with 38% aged 15–24. Nearly half (42%) had at least some secondary education; 87% identified as Christian and 12% as Muslim. 30% worked for pay within the last year. Overall, 43% were in severe poverty, with higher rates among SI users (52%) than DMPA-IM (43%) and provider-administered DMPA-SC users (34%).

Almost all had given birth (98%), averaging two children. More than a third did not want more children. 80% were married, and most partners were aware of the family planning visit.

10% were first-time family planning users. Nearly half (49%) had previously used injectables, and 11% had SI DMPA-SC before. Prior SI was reported by 9% of DMPA-IM users and 12% of provider-administered DMPA-SC users.

Injectables were the most recent method for 42% of participants (data not shown). Among these, prior use was lowest among SI users (37%) and highest among DMPA-IM users (46%). Mean duration of continuous injectable use was 43 months (SD 44), with DMPA-IM users reporting the longest duration (49 months; SD 48) and self-injectors and provider-administered DMPA-SC users reporting the shortest, both averaging 39 months (SD 45 and SD 36, respectively).

On the day of enrolment, 53% of provider-administered DMPA-SC users and 20% of DMPA-IM users received SI training; 67% of these non-SI users reported feeling comfortable with the training (data not shown).

Retention was high; 89% of the cohort was retained through the fourth follow-up (Web Only Table(s)/Web online supplemental appendix 1). No significant differences were found between those retained and those lost to follow-up (data not shown).

10.1136/bmjgh-2024-018761.supp1 Supplementary data

SI users typically received two DMPA-SC units per visit for future use. They were less likely to report transport costs to receive their method (18%–30%) compared to provider-administered DMPA-SC users (48%–78%) and DMPA-IM users (41%–61%). Similarly, missing work to receive their method was less common among SI users (7%–10%) than among provider-administered DMPA-SC (17%–25%) and DMPA-IM users (14%–30%).

### Research question #1 (IM vs PA SC): quantify any benefits related to user continuation

**Figure 1:**
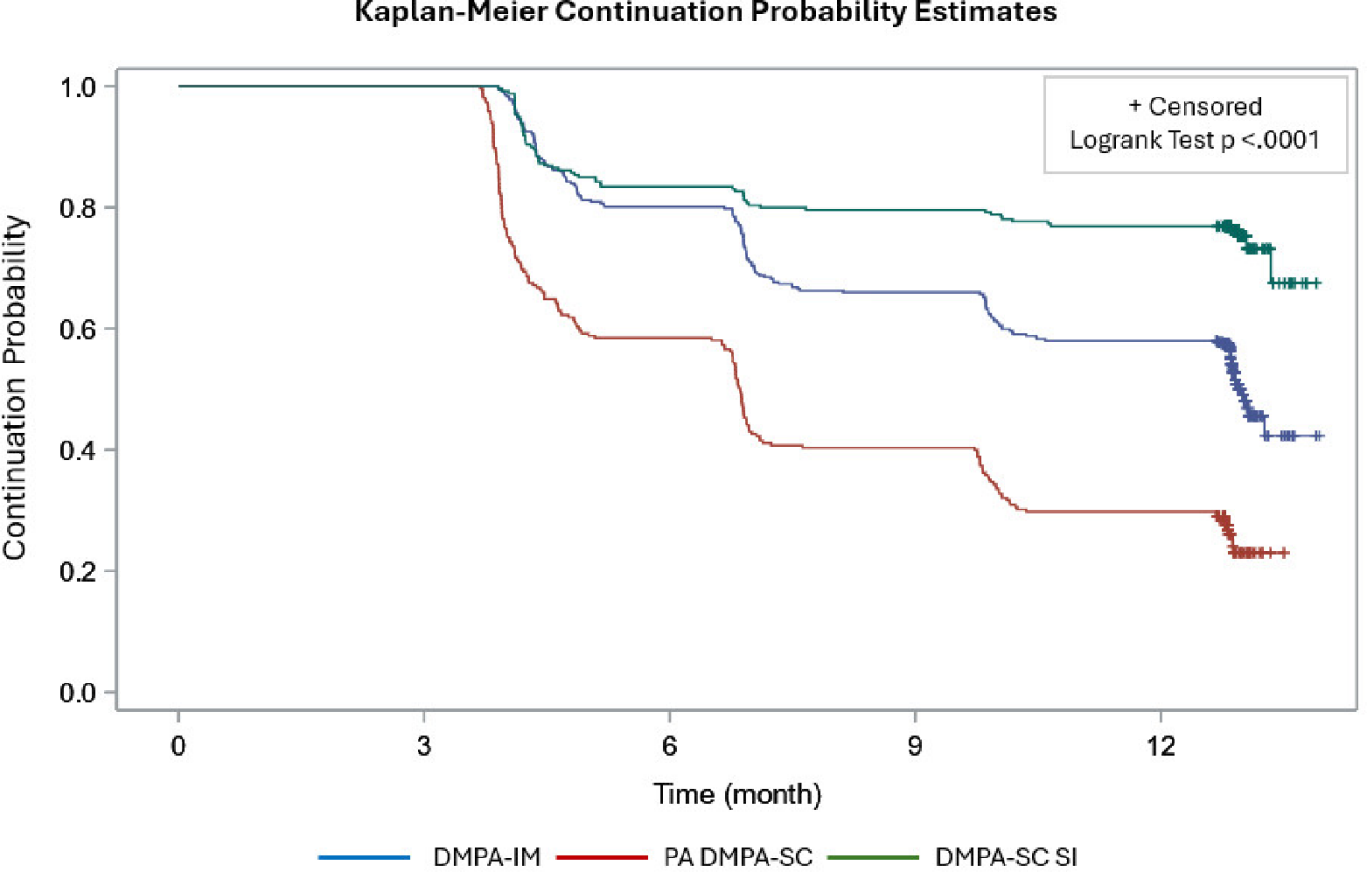
Kaplan-Meier probability curve: time to first method status change from enrolment method. DMPA-IM, depot medroxyprogesterone acetate-intramuscular; PA DMPA-SC, provider-administered depot medroxyprogesterone acetate subcutaneous; DMPA-SC SI, depot medroxyprogesterone acetate subcutaneous self-injection.

Continuation varied significantly by method (figure 1). SI users had the highest continuation, followed by DMPA-IM and then provider-administered DMPA-SC. Compared with DMPA-IM users, provider-administered DMPA-SC users were twice as likely to have a method status change (HR 2.01 (1.66–2.43)), while SI users were 39% less likely (HR 0.61 (0.48–0.77)).

Provider-administered DMPA-SC users transitioned to SI more frequently and faster than DMPA-IM users. At 12 months, the cumulative switching probability was 47.1% (95% CI 40.1 to 54.1%) for provider-administered DMPA-SC users compared with 20.4% (95% CI 16.0 to 24.9%) for DMPA-IM users. Those switching from provider-administered DMPA-SC did so in an average of 205 days versus 238 days for DMPA-IM users—typically at the second versus third reinjection visit, respectively.

Accounting for these transitions, the continuation gap between DMPA-IM and provider-administered DMPA-SC users narrowed from 25% to 6%, reflecting the impact of higher transition rates to SI among provider-administered DMPA-SC users.

### Research question #1 (IM vs PA SC): quantify any benefits to health system efficiencies

Data were collected through 23 group discussions with 141 DMPA service providers. These groups included nine with facility-based providers, nine with experienced HSAs and five with newly trained HSAs. Web Only Table(s)/Web online supplemental appendix 2 displays the inputs for the model comparing the costs for DMPA-IM to provider-administered DMPA-SC services.

10.1136/bmjgh-2024-018761.supp2 Supplementary data

Facility-based delivery of provider-administered DMPA-SC was more expensive than DMPA-IM, primarily due to higher labour costs, which made up 63% of the annual service costs (Web Only Table(s)/Web online supplemental appendix 3). Providers reported longer visit times with provider-administered DMPA-SC clients, often due to counselling on SI (though they did not self-inject).

10.1136/bmjgh-2024-018761.supp3 Supplementary data

Community-based services were less costly than facility-based services across all injectable options (Web Only Table(s)/Web online supplemental appendix 4 and Web Only Table(s)/Web online supplemental appendix 5). SI had the lowest delivery cost, even without factoring in extended pregnancy protection afforded by advanced provision at the follow-up visit.

10.1136/bmjgh-2024-018761.supp4 Supplementary data

10.1136/bmjgh-2024-018761.supp5 Supplementary data

The usage patterns, cost for DMPA services and cost per person-year of use are similar between the provider-administered DMPA-SC and DMPA-IM groups (table 2). Further, we found no substantial difference in annual DMPA service cost per person-year of use between DMPA-IM at US$11.01 (95% CI US$10.57–US$11.44) and PA DMPA-SC at US$11.47 (95% CI US$10.63–US$12.46).

**Table 2:** Annual DMPA service cost per person-year of use and to the healthcare system

Metric (95% CI from model)	PA DMPA-IM	PA DMPA-SC	Comparison PA DMPA-SC vs DMPA IM
Person-years of DMPA use	119 620 (108 046–133 108)	124 396 (104 722–144 617)	+4.0%
Annual DMPA service cost to healthcare system per 100 000 clients (USD)	$ 1 315 848 ($1 217 315–$1 415 628)	$ 1 422 534 ($1 256 641–$1 580 778)	+8.1%
Annual DMPA service cost per person-year of use (USD)	$ 11.01 ($10.57–$11.44)	$ 11.47 ($10.63–$12.46)	+4.0%

Note: the 95% CIs from simulated results overlapped for all comparisons.

DMPA-IM, provider-administered depot medroxyprogesterone acetate-intramuscular; PA DMPA-SC, provider-administered depot medroxyprogesterone acetate-subcutaneous.

Slightly more DMPA-IM users discontinued or switched methods compared with the provider-administered DMPA-SC cohort (4% difference on average, though this is not a substantial difference since the CIs overlap). Looking at the total costs of DMPA services, as expected, we see slightly higher costs in the provider-administered DMPA-SC cohort (8.1%) than the DMPA-IM cohort, though again the CIs overlap. We expect these costs to be higher due to more person-years of use by the cohort and higher commodity costs for DMPA-SC versus DMPA-IM. However, when we account for the difference in retention, the cost per person-year difference between the cohorts falls to 4% with a US$0.46 difference in cost per person-year, though again the CIs overlap. This difference is due to the higher commodity cost for DMPA-SC and slightly higher labour costs for provider-administered DMPA-SC compared with DMPA-IM (tables 3 and 4).

**Table 3:** Annual DMPA service cost per person-year of use and to the healthcare system

Metric (95% CI from model)	Facility-based services	Community-based services	Comparison community-based vs facility-based
Person-years of DMPA use	122 157 (104 643–140 519)	128 175 (105 727–150 236)	+4.9%
Annual DMPA service cost to healthcare system per 100 000 clients (USD)	$1 412 981 ($1 280 143–$1 549 501)	$1 028 146 ($878 039 –$1 180 035)	−27.2%*
Annual DMPA service cost per person-year of use (USD)	$11.61 ($10.70–$12.54)	$8.07 ($6.91–$9.41)	−30.7%*

*Non-overlapping 95% CIs from simulated results.

DMPA, depot medroxyprogesterone acetate.

**Table 4:** DMPA service cost breakdown per person-year protection by DMPA option (USD)

DMPA option	Facility-based	Community-based	% difference
Overall	$11.57	$8.02	−30.7%
DMPA-IM	$11.56	$11.36	−1.7%
Provider-administered DMPA-SC	$13.81	$14.05	+1.8%
Self-injection	$10.35	$6.30	−39.1%

DMPA-IM, depot medroxyprogesterone acetate-intramuscular; DMPA-SC, depot medroxyprogesterone acetate-subcutaneous.

A key efficiency gain came from transitions to SI. For users who made the transition to self-injection, there is a large saving to the healthcare system as healthcare labour costs fall to zero for subsequent SI by the clients until there is a need for resupply. Among provider-administered DMPA-SC users, SI accounted for 43.4% of person-years and 28.7% of total costs, reducing labour costs to zero for those periods. In contrast, DMPA-IM users were less likely to switch, with DMPA-IM accounting for 71.9% of person-years and 77% of total costs (Web Only Table(s)/Web online supplemental appendix 6).

10.1136/bmjgh-2024-018761.supp6 Supplementary data

### Research question #2: what is the ‘value proposition’ of community-based distribution?

While the usage patterns were similar, annual DMPA service costs were 27% higher for facility-based services than community-based services (table 3). In addition, the cost per person-year was 31% lower in community settings—US$8.07 (US$6.91–US$9.41) versus US$11.61 (US$10.70–US$12.54) for facility-based services.

Community-based services offered comparable contraceptive protection at a lower cost. Differences between service locations were modest for DMPA-IM and provider-administered DMPA-SC but substantial for SI (table 4). Cost savings in community settings were driven by lower labour costs and shorter visit durations reported by HSAs compared with facility-based staff.

### Breakeven analysis

The models demonstrate that the absolute DMPA service costs of provider-administered DMPA-SC are the most expensive option on a per-client basis, although not substantially different from the DMPA-IM group. This is due to the higher commodity cost of DMPA-SC than DMPA-IM and the longer provider contact time so that providers may counsel clients about the DMPA-SC formulation and the potential for SI. This raises the question of how much of DMPA-SC use needs to be for SI for DMPA-SC to become less expensive, overall, than DMPA-IM. Web Only Table(s)/Web online supplemental appendix 7 and Web Only Table(s)/Web online supplemental appendix 8 show how the cost of DMPA-SC services falls as the proportion of SI use increases and the point where it becomes equivalent to DMPA-IM on an annual cost per client basis.

10.1136/bmjgh-2024-018761.supp7 Supplementary data

10.1136/bmjgh-2024-018761.supp8 Supplementary data

For facility-based services, delivering DMPA-SC is less expensive compared with DMPA-IM when 40% or more of all DMPA-SC visits are for SI, whereas for community-based services, only 23% of DMPA-SC visits need to be for SI for DMPA-SC to be cost-saving.

Service statistics from the study catchment areas show that SI rates have consistently exceeded these thresholds. Between January and June 2024, 64%–70% of DMPA-SC visits were for SI, indicating that DMPA-SC is already cost-saving in these settings.

## Discussion

In this study in central and southern Malawi, we observed more provider-administered DMPA-SC users transitioning to SI and transitioning faster to SI compared with DMPA-IM users, which highlights the attractiveness of SI to clients who gain exposure to the DMPA-SC product. When considering differences in user experience, transition and contraceptive method failure rates through a 12-month period, we also found no substantial difference in the annual DMPA service cost per person-year of use between DMPA-IM and provider-administered DMPA-SC. We found potentially even greater cost-savings when services are provided in community settings. Routine injectable service delivery data that we collected from the study catchment areas during the study indicate that breakeven thresholds for the proportion of DMPA-SC that needs to be for SI are achievable. We followed the cohort for only 1 year, but if those who transitioned to SI continue to self-inject, the cost savings will only increase.^11–13^ Further, when a generic DMPA-SC becomes available, the price of DMPA-SC will decrease, and the cost savings will increase. While our results should be confirmed in other settings, this study demonstrated that family planning programmes may not need all DMPA-SC users to self-inject for DMPA-SC to be cost-competitive with DMPA-IM, and programmes may benefit from offering the provider-administered option, which may increase the probability of switching to SI over time.

Our results are consistent with previous studies. Similar to our findings, the study in Burkina Faso, Uganda and Senegal found the costs were similar for DMPA-IM and DMPA-SC when administered by the same type of health worker in the same setting.^10 11^ The annual DMPA service costs we calculated in Malawi for DMPA-IM at US$11.01 and provider-administered DMPA-SC at US$11.47 were similar to the costs calculated in the previous study for facility-based delivery of DMPA-IM (US$10.12 in Uganda; US$9.46 in Senegal; US$11.60 in Burkina Faso) and provider-administered DMPA-SC in Burkina Faso (US$12.14). Also, like our Malawi study, the previous study found that the costs of community-based delivery of DMPA-IM and DMPA-SC (US$7.71 and US$7.69) were lower compared with facility-based delivery of DMPA-IM in Uganda.^11^ Further, the previous study found that the largest cost component in provider-administered injectable services is the cost of provider time, which is consistent with our findings and adds further support for expanding community-based delivery of family planning, including all options of injectables.^11^

Our study builds on the previous studies because it was conducted outside of pilot settings and therefore measured costs during routine service delivery conditions. In addition, our modelling approach was more dynamic than past studies. Past studies measured continuation status at 12 months and used a static model that assumed discontinuation happened at 6 months and that women who continued for the year used four units of DMPA.^11–13^ In contrast, we measured family planning method use every 3 months and incorporated observed method switching and discontinuation behaviours measured over the 12 months into the models, as well as the reported number of DMPA-SC units given to self-injectors to take home.

Our study had some limitations worth noting. Like the other studies, our study did not include costs for setting up a community-based distribution programme, training and supervising providers to deliver injectables, facility operational and management costs, or supply chain costs. Our models did not include costs for the treatment of side effects or health facility waste disposal, though previous studies did not find these factors to be significant cost drivers.^11^ The costing models we present only focus on the DMPA service costs, which take into account the rate of discontinuation of DMPA products but not the costs of using non-DMPA products. While we measured the non-DMPA products cohort participants switched to, and their costs for using family planning methods throughout the study, to keep the models simple, we did not include these costs in the models that we present here. In a future paper, we will report on models from the societal perspective, which account for the full method use dynamics observed in our cohort study and consider the costs associated with use of other methods and the costs of unintended pregnancies, as well as the costs incurred by the participants. As with any modelling exercise, our models use simplifying assumptions. Despite these limitations, our models are more realistic and extend beyond those of previous studies by incorporating switching to and from SI over 12 months and using this information to estimate the breakeven point for DMPA-SC. This was not possible before since SI was not routinely available in the countries at the time of previous studies.^11–13^

SI of DMPA-SC has clear advantages for users over provider-administered DMPA^4–6^ and is the most cost-effective use of DMPA.^10–13^ However, not all injectable users will want to immediately self-inject, and some will never want to self-inject.^14^ Our team’s previous qualitative research in Malawi found that some clients prefer to be eased into SI by having the provider inject DMPA-SC for them at first, and over time they become more confident to self-inject.^16^ These qualitative findings are supported by our current quantitative study, which suggests that provider-administered DMPA-SC may increase the uptake of SI over time.

Based on the study findings, we recommend that in addition to offering SI, countries allow for the provider-administered DMPA-SC option in both facilities and in communities because offering clients provider-administered DMPA-SC is an important stepping stone to SI without any substantial additional cost and, importantly, expands women’s choices, respecting their individual autonomy and supporting their reproductive rights.

10.1136/bmjgh-2024-018761.supp9 Supplementary data

## Data Availability

Data are available upon reasonable request.

## References

[1] National Statistical Office . Malawi multiple indicator cluster survey 2019-20, survey findings report. Zomba, Malawi National Statistical Office; 2021.

[2] Burke HM , Mueller MP , Perry B , et al . Observational study of the acceptability of Sayana® Press among intramuscular DMPA users in Uganda and Senegal. Contraception 2014;89:361–7. 10.1016/j.contraception.2014.01.022 24631328

[3] Burke HM , Mueller MP , Packer C , et al . Provider acceptability of Sayana® Press: results from community health workers and clinic-based providers in Uganda and Senegal. Contraception 2014;89:368–73. 10.1016/j.contraception.2014.01.009 24576792

[4] Burke HM , Chen M , Buluzi M , et al . Effect of self-administration versus provider-administered injection of subcutaneous depot medroxyprogesterone acetate on continuation rates in Malawi: a randomised controlled trial. Lancet Glob Health 2018;6:e568–78. 10.1016/S2214-109X(18)30061-5 29526707

[5] Kennedy CE , Yeh PT , Gaffield ML , et al . Self-administration of injectable contraception: a systematic review and meta-analysis. BMJ Glob Health 2019;4:e001350. 10.1136/bmjgh-2018-001350 PMC6528768 31179026

[6] Cover J , Namagembe A , Tumusiime J , et al . Continuation of injectable contraception when self-injected vs. administered by a facility-based health worker: a nonrandomized, prospective cohort study in Uganda. Contraception 2018;98:383–8. 10.1016/j.contraception.2018.03.032 29654751 PMC6197833

[7] Pfizer . Sayana® press label. Available: https://labeling.pfizer.com/ShowLabeling.aspx?id=15149 [Accessed 06 May 2025].

[8] PATH . DMPA-SC Resource Library, Available: https://fpoptions.org/about/ [Accessed 06 May 2025].

[9] Rademacher KH , Solomon M , Brett T , et al . Expanding Access to a New, More Affordable Levonorgestrel Intrauterine System in Kenya: Service Delivery Costs Compared With Other Contraceptive Methods and Perspectives of Key Opinion Leaders. Glob Health Sci Pract 2016;4 Suppl 2:S83–93. 10.9745/GHSP-D-15-00327 27540128 PMC4990165

[10] PATH . Costs and cost-effectiveness of DMPA-SC. Available: https://fpoptions.org/wp-content/uploads/DMPA-SC-advopack-handout-cost-effectiveness-PATH-2023.pdf [Accessed 06 May 2025].

[11] Di Giorgio L , Mvundura M , Tumusiime J , et al . Costs of administering injectable contraceptives through health workers and self-injection: evidence from Burkina Faso, Uganda, and Senegal. Contraception 2018;98:389–95. 10.1016/j.contraception.2018.05.018 29859148 PMC6197836

[12] Di Giorgio L , Mvundura M , Tumusiime J , et al . Is contraceptive self-injection cost-effective compared to contraceptive injections from facility-based health workers? Evidence from Uganda. Contraception 2018;98:396–404. 10.1016/j.contraception.2018.07.137 30098940 PMC6197841

[13] Mvundura M , Di Giorgio L , Morozoff C , et al . Cost-effectiveness of self-injected DMPA-SC compared with health-worker-injected DMPA-IM in Senegal. Contracept X 2019;1:100012. 10.1016/j.conx.2019.100012 32494776 PMC7252428

[14] Wood SN , Magalona S , Zimmerman LA , et al . Self-injected contraceptives: does the investment reflect women’s preferences? BMJ Glob Health 2022;7:e008862. 10.1136/bmjgh-2022-008862 PMC9289037 35835480

[15] Tariq F , Swearingen R . DISC empathy training evaluation study findings. 2024. Available: https://media.psi.org/wp-content/uploads/2024/02/16135340/DISC-Empathy-Training-Evaluation-Study-Findings.pdf [Accessed 06 May 2025].

[16] Burke HM , Packer C , Zingani A , et al . Testing a counseling message for increasing uptake of self-injectable contraception in southern Malawi: A mixed-methods, clustered randomized controlled study. PLoS ONE 2022;17:e0275986. 10.1371/journal.pone.0275986 36256638 PMC9578616

[17] Burke HM , Packer C , Buluzi M , et al . Client and provider experiences with self-administration of subcutaneous depot medroxyprogesterone acetate (DMPA-SC) in Malawi. Contraception 2018;98:405–10. 10.1016/j.contraception.2018.02.011 29706227

[18] Burke HM , Packer C , Wando L , et al . Adolescent and covert family planning users’ experiences self-injecting contraception in Uganda and Malawi: implications for waste disposal of subcutaneous depot medroxyprogesterone acetate. Reprod Health 2020;17:117. 10.1186/s12978-020-00964-1 32746860 PMC7396890

[19] Ross J , Stover J . Use of modern contraception increases when more methods become available: analysis of evidence from 1982-2009. Glob Health Sci Pract 2013;1:203–12. 10.9745/GHSP-D-13-00010 25276533 PMC4168565

[20] Injectables Access Collaborative . Phases of market development for DMPA-SC self-injection: framework and country status as of Q2 2024. PATH; 2024.

[21] Injectables Access Collaborative . Global DMPA status report 2024 Q2. PATH; 2024.

[22] Wynne L , Fischer S . Malawi’s self-care success story: rapid introduction of self-injectable contraception, 2020. Available: https://knowledgesuccess.org/2020/07/22/malawis-self-care-success-story-rapid-introduction-of-self-injectable-contraception/ [Accessed 06 May 2025].

[23] PSI . Delivering innovation in self-care (DISC). Available: https://www.psi.org/disc/home/ [Accessed 06 May 2025].

[24] Cutherell A , Nakanwagi A , Odeh R , et al . Overcoming client fear to self-inject: consumer insights light a path toward DMPA-SC scale-up. PSI. Available: https://www.psi.org/project/disc/overcoming-client-fear-to-self-inject-dmpa-sc-scale-up/ [Accessed 06 May 2025].

[25] Lakatos E . Sample sizes based on the log-rank statistic in complex clinical trials. Biometrics 1988;44:229–41. 3358991

[26] NCSS L . Power analysis and sample size software. Kaysville, Utah, USA, 2022.

[27] Guest G , Namey E , McKenna K . How Many Focus Groups Are Enough? Building an Evidence Base for Nonprobability Sample Sizes. Field methods 2017;29:3–22. 10.1177/1525822X16639015

[28] Ayer Company Publishers, Inc . Contraceptive Technology. New York, NY, 2018.

[29] Singh S , Darroch JE , Ashford LS . The costs and benefits of investing in sexual and reproductive health 2014. Guttmacher Institute; 2014.

[30] Oxford Poverty and Human Development Initiative (OPHI) . What is the global MPI. Available: https://ophi.org.uk/what-global-mpi [Accessed 06 May 2025].

