# Supplementary figures and images for: The case for investing in provider-administered subcutaneous DMPA: a costing study
Source: BMJ Glob Health. 2025 Oct 22;10(Suppl 6):e018761. doi: 10.1136/bmjgh-2024-018761 (PMC12826344; doi:10.1136/bmjgh-2024-018761)

Web Only Table(s)/Web Appendix 1. Disposition chart

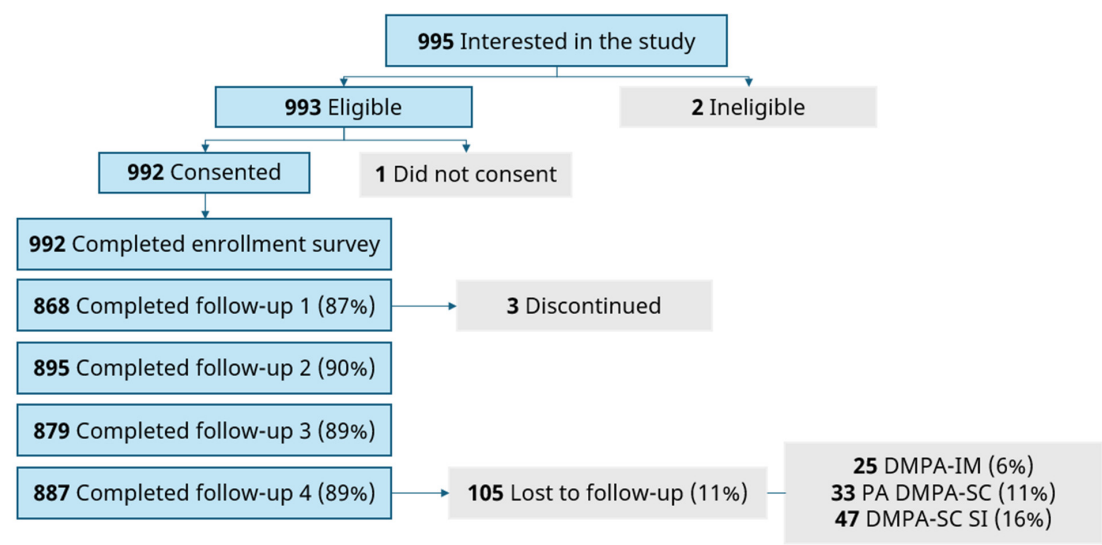

Supplement: Supplementary data [file bmjgh-10-Suppl_6-s001.pdf]

Web Only Table(s)/Web Appendix 7. Break-even analysis for health facility-based services

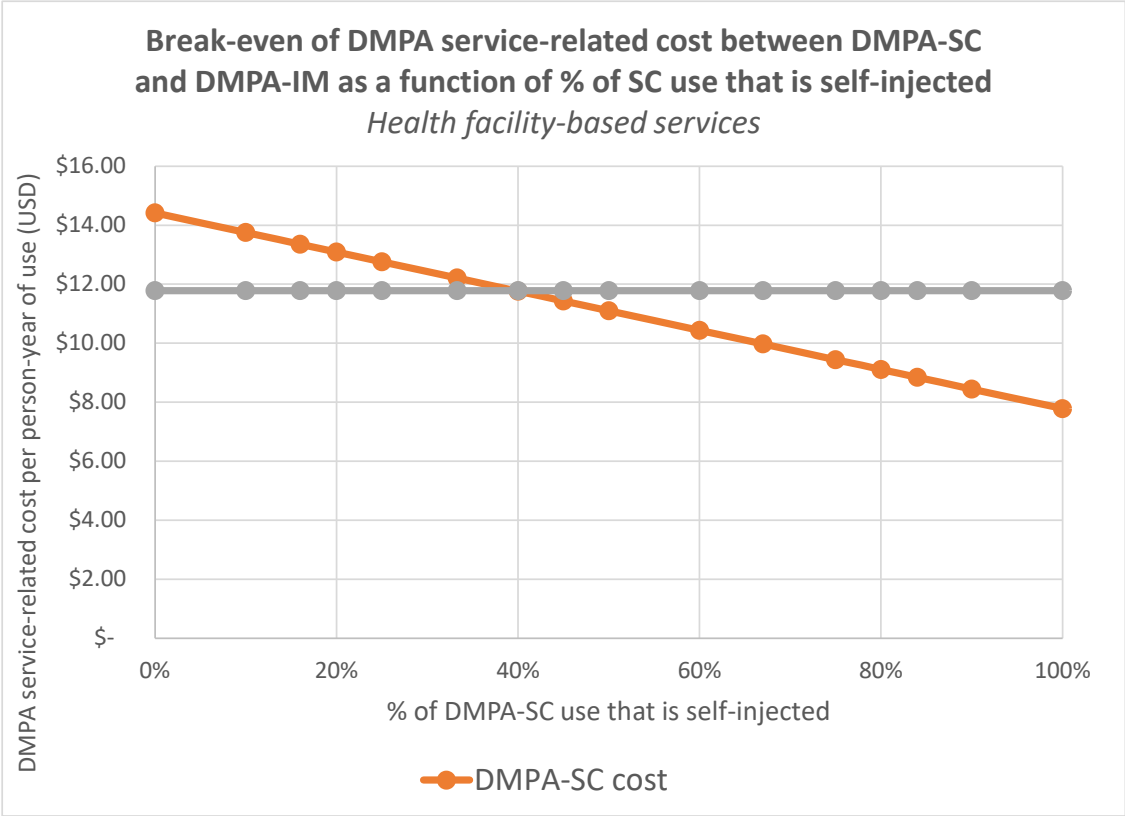

Supplement: Supplementary data [file bmjgh-10-Suppl_6-s007.pdf]

Web Only Table(s)/Web Appendix 8. Break-even analysis for community-based services

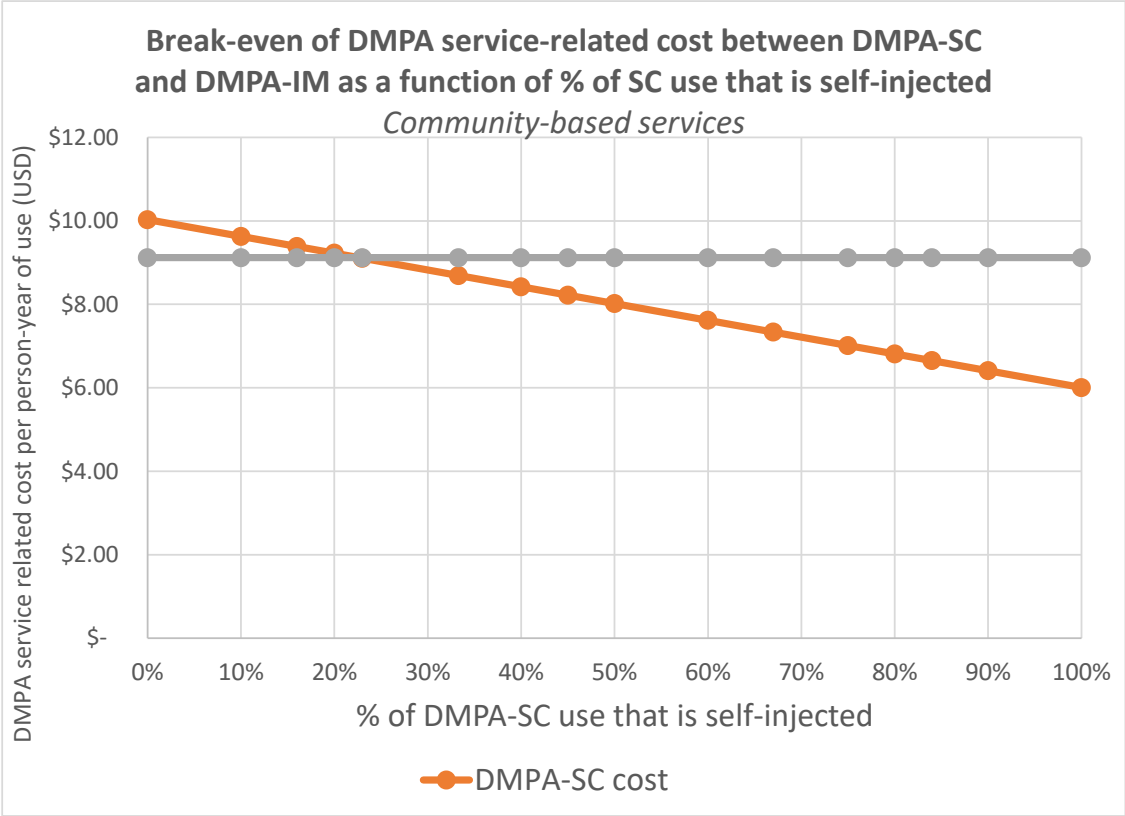

Supplement: Supplementary data [file bmjgh-10-Suppl_6-s008.pdf]
