# Supplementary material for: The case for investing in provider-administered subcutaneous DMPA: a costing study
Source: BMJ Glob Health. 2025 Oct 22;10(Suppl 6):e018761. doi: 10.1136/bmjgh-2024-018761 (PMC12826344; doi:10.1136/bmjgh-2024-018761)
Supplement: Supplementary data [file bmjgh-10-Suppl_6-s002.pdf]

Web Only Table(s)/Web Appendix 2.

Inputs for models of DMPA service costs within the health care system

Inputs	DMPA-IM	PA DMPA-SC	Source
Discontinuation rate:			Cohort study: participant method status over time.
1-3 months	0.095	0.186	
4-6 months	0.107	0.081	
7-9 months	0.105	0.108	
10-12 months	0.114	0.098	
Proportion switch to/stay on DMPA-IM:			Cohort study: participant method status over time.
1-3 months	0.796	0.059	
4-6 months	0.700	0.063	
7-9 months	0.619	0.063	
10-12 months	0.587	0.064	
Proportion switch to/stay on PA DMPA-SC:			Cohort study: participant method status over time.
1-3 months	0.026	0.569	
4-6 months	0.051	0.453	
7-9 months	0.056	0.353	
10-12 months	0.073	0.346	
Proportion switch to SI:			Cohort study: participant method status over time.
1-3 months	0.082	0.186	
4-6 months	0.142	0.403	
7-9 months	0.220	0.476	
10-12 months	0.226	0.492	
Cost of method initiation:			Resource requirements focus group discussions; Guttmacher estimates [27].
Time 0 & 1	$3.55	$4.40	
Time 2	$3.58	$4.38	
Time 3	$3.55	$4.37	

Time 4	$3.55	$4.41	
Cost of method continuation:<br>Time 1<br>Time 2<br>Time 3<br>Time 4	 $2.70<br>$2.72<br>$2.69<br>$2.68	 $3.23<br>$3.27<br>$3.26<br>$3.30	
Cost of SI use†:<br>Initiation<br>Continuation	 $6.04<br>$4.91	 $6.04<br>$4.91	
Cost of method discontinuation	$0.00	$0.00	No cost to discontinue DMPA Use.
DMPA-IM and DMPA-SC failure rates:<br>1-3 months<br>4-6 months<br>7-9 months<br>10-12 months	 0.0042<br>0.0042<br>0.0047<br>0.0047	 0.0042<br>0.0042<br>0.0047<br>0.0047	Contraceptive Technology publication [26]. Value based upon exponentiation of the annual failure rate under typical use to 0.25 to represent a quarterly risk.

†SI visit costs include advanced provision of two additional DMPA-SC units based on cohort study findings. SI users in the models receive nine months of pregnancy protection for every in-person FP visit (one injection on the day of the FP visit and two more through advanced provision).

Distribution of 100,000 PA DMPA-IM users at baseline

Product Used follow-up	Time 1 (n)	Time 2 (n)	Time 3 (n)	Time 4 (n)
PA DMPA-IM	74,450	63,601	54,134	50,952
PA DMPA-SC	3,460	6,538	7,252	9,260
SI DMPA-SC	10,238	16,591	25,807	25,652

Distribution of 100,000 PA DMPA-SC users at baseline

Product Used follow-up	Time 1 (n)	Time 2 (n)	Time 3 (n)	Time 4 (n)
PA DMPA-IM	7,222	8,374	8,287	8,217
PA DMPA-SC	48,558	39,941	30,270	29,575
SI DMPA-SC	21,546	41,419	48,107	50,023
