# Supplementary material for: The case for investing in provider-administered subcutaneous DMPA: a costing study
Source: BMJ Glob Health. 2025 Oct 22;10(Suppl 6):e018761. doi: 10.1136/bmjgh-2024-018761 (PMC12826344; doi:10.1136/bmjgh-2024-018761)
Supplement: Supplementary data [file bmjgh-10-Suppl_6-s003.pdf]

Web Only Table(s)/Web Appendix 3. Facility-based service delivery costs for provider-administered injectables

	Facility-based					
Option	*DMPA-IM*			*PA DMPA-SC*		
**Cost Component**	**Initial Visit**	**Revisit**	**Annual Cost^**	**Initial Visit**	**Revisit**	***Annual Cost^***
Commodity	$ 0.85	$ 0.85	$ 3.40 (29%)	$ 1.05	$1.05	$ 4.20 (29%)
Supplies	$ 0.43	$ 0.43	$ 1.72 (15%)	$ 0.26	$ 0.26	$ 1.04 (7%)
Labor	$ 2.29	$ 1.43	$ 6.58 (56%)	$ 3.10	$ 2.00	$ 9.10 (63%)
Infrastructure	$ 0.02	$ 0.02	$ 0.08 (1%)	$ 0.02	$ 0.02	$ 0.08 (1%)
Total	*$ 3.59*	*$ 2.73*	***$ 11.78 (100%)***	*$4.43*	*$3.33*	***$14.42 (100%)***

^ Assumes one initial visit plus three revisits per calendar year
