# Supplementary material for: The case for investing in provider-administered subcutaneous DMPA: a costing study
Source: BMJ Glob Health. 2025 Oct 22;10(Suppl 6):e018761. doi: 10.1136/bmjgh-2024-018761 (PMC12826344; doi:10.1136/bmjgh-2024-018761)
Supplement: Supplementary data [file bmjgh-10-Suppl_6-s004.pdf]

Web Only Table(s)/Web Appendix 4. Community-based service delivery costs for provider-administered injectables

	Community-based					
Option	*DMPA-IM*			*PA DMPA-SC*		
**Cost Component**	**Initial Visit**	**Revisit**	**Annual Cost^**	**Initial Visit**	**Revisit**	***Annual Cost^***
Commodity	$ 0.85	$ 0.85	$ 3.40 (37%)	$ 1.05	$ 1.05	$ 4.20 (42%)
Supplies	$ 0.43	$ 0.43	$ 1.72 (19%)	$ 0.35†	$ 0.26	$ 1.13 (11%)
Labor	$ 1.72	$ 0.76	$ 4.00 (44%)	$ 2.00	$ 0.90	$ 4.70 (47%)
Infrastructure	-	-	-	-	-	-
Total	*$ 3.00*	*$ 2.04*	***$ 9.12 (100%)***	*$3.40*	*$2.21*	***$10.03 (100%)***

^ Assumes one initial visit plus three revisits per calendar year

† At initial visit, PA DMPA-SC users were given an info sheet which was not given at revisits.
