# Supplementary material for: The case for investing in provider-administered subcutaneous DMPA: a costing study
Source: BMJ Glob Health. 2025 Oct 22;10(Suppl 6):e018761. doi: 10.1136/bmjgh-2024-018761 (PMC12826344; doi:10.1136/bmjgh-2024-018761)
Supplement: Supplementary data [file bmjgh-10-Suppl_6-s005.pdf]

Web Only Table(s)/Web Appendix 5. DMPA-SC self-injection service delivery costs by service delivery location

	DMPA-SC Self-injection							
Option	*Facility-based*				*Community-based*			
**Cost Component**	**Initial Visit**	**Revisit**	**Annual Cost***	**Cost/ person-year†**	**Initial Visit**	**Revisit**	**Annual Cost***	**Cost/ person-year†**
Commodity^	$3.15	$3.15	$6.30 (58%)	$4.20 (58%)	$3.15	$3.15	$6.30 (70%)	$4.20 (70%)
Supplies	$0.35 ‡	$0.26	$0.61 (6%)	$0.41 (6%)	$0.35‡	$0.26	$0.61 (7%)	$0.41 (7%)
Labor	$3.14	$1.59	$4.73 (44%)	$3.15 (44%)	$1.23	$0.87	$2.10 (23%)	$1.40 (23%)
Infrastructure	$0.02	$0.02	$0.04 (0%)	$0.03 (0%)	-	-	-	-
Total	*$6.66*	*$ 4.21*	***$10.87 (100%)***	***$7.25 (100%)***	*$4.73*	*$4.28*	***$9.01 (100%)***	***$6.01 (100%)***

^ Assumes two additional units given to the client at each visit for subsequent use
* Assumes one initial visit and one revisit at ~ 11 months
† With 6 units total given per client per year, annual cost yields 1.5 person-years of protection
‡ At initial visit, self-injectors were given an info sheet which was not given at revisits.
