# Supplementary material for: The case for investing in provider-administered subcutaneous DMPA: a costing study
Source: BMJ Glob Health. 2025 Oct 22;10(Suppl 6):e018761. doi: 10.1136/bmjgh-2024-018761 (PMC12826344; doi:10.1136/bmjgh-2024-018761)
Supplement: Supplementary data [file bmjgh-10-Suppl_6-s006.pdf]

Web Only Table(s)/Web Appendix 6. Breakdown of costs and person-years of use within the cohort groups

	PA DMPA-IM Cohort	DMPA-SC Cohort
% of Costs from DMPA-IM use	77.0%	6.2%
% of Costs from PA DMPA-SC use	7.4%	65.1%
% of Costs from DMPA-SC SI use	15.6%	28.7%
% of person-years from DMPA-IM use	71.9%	6.5%
% of person-years from PA DMPA-SC use	5.6%	50.2%
% of person-years from DMPA-SC SI use	22.6%	43.4%
