# Supplementary material for: The case for investing in provider-administered subcutaneous DMPA: a costing study
Source: BMJ Glob Health. 2025 Oct 22;10(Suppl 6):e018761. doi: 10.1136/bmjgh-2024-018761 (PMC12826344; doi:10.1136/bmjgh-2024-018761)
Supplement: Supplementary data [file bmjgh-10-Suppl_6-s009.pdf]

**Appendix S1 – Reflexivity Statement**

**1. How does this study address local research and policy priorities?**

This research study and resulting manuscript was specifically designed to inform Malawi's national family planning program. This is also an issue of concern from other governments in low- and middle-income countries who are considering or have introduced DMPA-SC and self-injection into their family planning method mix.

**2. How were local researchers involved in study design?**

The first category of local researchers involved are those with experience implementing research in Malawi (WFN, HK, and GJ). These co-authors provided direction for the data collection methods based on their experiences conducting similar research studies in Malawi. WFN, HK, and GJ also provided input into the design of the data collection tools and other study forms (i.e., consent forms, recruitment scripts), in addition to contributing to the interpretation and reporting of the study findings. WFN made numerous presentations about the study to Malawi family planning stakeholders throughout the project. Specifically, WFN led the efforts to obtain local feedback on the study design and methods during the planning phase in addition to disseminating the preliminary and final study results in Malawi. WFN served as the site principal investigator for the study.

The second category is a local researcher who works for the Government of Malawi's Reproductive Health Directorate within the Ministry of Health (JSC). In her role, JSC has extensive knowledge about and influence on Malawi's family planning program. JSC served as a co-investigator and contributed to the design of the study including the approach, methods, and study site locations.

**3. How has funding been used to support the local research team?**

This study fully funded the first category of researchers (WFN, HK, and GJ) to collect the data for the study, as well as for their time contributing to the design, analysis, and reporting of the study results. Additionally, the first category of researchers was able to use funding from this study to attend Kamuzu University of Health Sciences' Research Dissemination Conference ($2^{nd}$ – $4^{th}$ October 2024 in Blantyre, Malawi), as an opportunity for professional development

**4. How are research staff who conducted data collection acknowledged?**

The research staff who were responsible for collecting the data (WFN, HK, and GJ) were included as authors.

**5. Do all members of the research partnership have access to study data?**

All members of the partnership have access to data.

**6. How was data used to develop analytical skills within the partnership?**

All members of the partnership were involved in reviewing and interpreting the data.

**7. How have research partners collaborated in interpreting study data?**

All members of the partnership reviewed the study results and participated in calls spanning across several months to discuss meaning of the results and the recommendations that flow from the results. We also discussed how to present the findings and recommendations to maximize their utilization and impact. All partners collaborated to agree on the recommendations stemming from the study findings and contents of this reflexivity statement.

**8. How were research partners supported to develop writing skills?**

While the research team members who wrote the first draft of the manuscript are high-income country researchers, our Malawi-based colleagues and co-authors reviewed multiple drafts of the manuscript and the other research products such as PowerPoint presentations and provided multiple rounds of feedback to improve the products.

**9. How will research products be shared to address local needs?**

WFN disseminated the study results to Malawian family planning stakeholders throughout the project including preliminary and final study results. WFN also presented the study results at a Malawian academic conference.

**10. How is the leadership, contribution and ownership of this work by LMIC researchers recognised within the authorship?**

WFN's contributions to the research have been recognized with second authorship, a position we consider significant. We acknowledge, however, that the first and last authors are from a high-income country. This authorship order primarily reflects their substantial contributions to the study's conceptualization, funding acquisition, design, and manuscript writing.

11. **How have early career researchers across the partnership been included within the authorship team?**

We have included early career researchers from Malawi (HK and GJ) within the authorship team.

**12. How has gender balance been addressed within the authorship?**

Five authors are female (HMB, MML, JSC, HK, and GJ) and three authors male (WFN, RH and MC).

**13. How has the project contributed to training of LMIC researchers?**

Authors HK and GJ gained experience conducting group discussions and synthesizing results from those discussions because of their work during this study. HK and GJ gained further project management and supervision experience and the opportunity to present at a conference for the first time. WFN, HK and GJ were given support and coaching in communicating the findings so they could lead local dissemination efforts with confidence.

**14. How has the project contributed to improvements in local infrastructure?**

This project has not directly contributed to improvements in local infrastructure.

**15. What safeguarding procedures were used to protect local study participants and researchers?**

This study was reviewed and approved by the National Health Sciences Research Committee (NHSRC) in Malawi (protocol # 23/03/3196) and FHI 360's Protection of Human Subjects Committee (PHSC) (protocol # 2015937) and conforms to the principles embodied in the Declaration of Helsinki. All participants provided informed consent prior to participating in the study. Research assistants were trained to ensure the privacy and confidentiality of participants and staff completed a safe guarding training. We have specifically reported on safeguarding within the manuscript.
